# Bisdemethoxycurcumin inhibits ovarian cancer via reducing oxidative stress mediated MMPs expressions

**DOI:** 10.1038/srep28773

**Published:** 2016-06-28

**Authors:** Haifeng Pei, Yi Yang, Lin Cui, Jiong Yang, Xiuchuan Li, Yongjian Yang, Haixia Duan

**Affiliations:** 1 Department of Cardiology, Chengdu Military General Hospital, Chengdu 610083, China; 2 Department of Orthopedics, Chengdu Military General Hospital, Chengdu 610083, China; 3 Department of Obstetrics and Gynecology, Affiliated Guangren Hospital, College of Medicine, Xi’an Jiaotong University, Xi’an 710004, China

## Abstract

As one main active compound of curcuminoids, Bisdemethoxycurcumin (BDMC) possesses several biological activities, such as anti-inflammation and anti-cancer activities. However, the detailed mechanism of BDMC’s anti-metastasis activity in ovarian cancer has not been clearly elucidated yet. In the present study, cell proliferation, wound healing motility, cell adhesion and invasion with or without BDMC were determined. In addition, western blot was used to examine proteins expressions. The lucigenin-enhanced luminescence was introduced to assess cellular oxidative stress. The luciferase reporter gene assay was introduced to evaluate the transcriptional activity of NF-κB. Finally, BDMC significantly inhibited the adhesion, migration, invasion and metastasis of SKOV-3 cells. Moreover, BDMC inhibited expressions of several degradation-associated proteins, such as matrix metalloproteinase-2 (MMP-2), matrix metalloproteinase-9 (MMP-9), CD147, urokinase plasminogen activator (uPA), intercellular adhesion molecule-1 (ICAM-1) and vascular cell adhesion molecule-1 (VCAM-1), whereas increased expression of tissue inhibitor of metalloproteinase-1 (TIMP-1), in a dose-dependent manner. In addition, BDMC reduced generation of cellular superoxide in a dose-dependent manner. Furthermore, BDMC inhibited the phosphorylation levels of NF-κB p65 and IκB-α, and consequently reduced NF-κB-driven luciferase expression. Collectively, BDMC serves as a therapeutic medicine to suppress ovarian cancer, perhaps via inhibiting cellular oxidative stress and subsequently inactivating NF-κB pathway.

As the most frequent cause of death from gynecologic neoplasms all around the world, ovarian carcinoma has become a crucial health problem. Due to the severe invasion and metastasis, ovarian carcinoma’s overall 5-year survival is only 30%. In view of the rising overall incidence and mortality, we should develop new effective approaches to prevent and treat this disease.

Tumor metastasis includes cell adhesion, invasion, and angiogenesis, etc., in which extracellular matrix (ECM) degradation induced by proteolytic enzymes is a crucial step^1^. As far as we know, matrix metalloproteinase (MMPs) and urokinase plasminogen activator (uPA) are two main enzymes to degrade ECM components. Notably, MMPs [such as MMP-2 (gelatinase A) and MMP-9 (gelatinase B)] have been reported to degrade type IV collagen to facilitate cancer cell invasion and metastasis^2^. Moreover, MMPs secretion can be influenced by NADPH oxidases mediated reactive oxygen species (ROS) generation in human articular chondrocytes^3^. Verma *et al*.^4^ also reveal that oxidative stress is closely involved in cancer by changing the activity and expression of regulatory proteins, especially MMPs. Thus, ROS overload may increase MMPs secretion to enhance ECM degradation, consequently aggravating ovarian cancer. Furthermore, ROS generation is able to activate NF-κB signaling^5^, thus NF-κB may serve as an important effector downstream of oxidative stress in ovarian cancer.

In view of the urgent need to cope with ovarian cancer, several natural agents have been paid attention gradually to assess the anti-cancer efficiency. As a natural phenolic compound extracted from curcuma species’ rhizome^6^, Bisdemethoxycurcumin (BDMC) possesses important biological properties against inflammatory, oxidative stress, and angiogenesis^7^,^8^,^9^. However, the detailed effects of BDMC on human ovarian cancer SKOV-3 cells have not been fully studied, no matter the underlying molecular mechanisms.

## Materials and Methods

### Reagents

BDMC (Sigma, USA) was dissolved in dimethyl sulfoxide (DMSO) at a concentration of 10 mM (stored in a dark-colored bottle at −20 °C as a stock solution). Rabbit anti-human uPA, Rabbit anti-human MMP-2/9, Rabbit anti-humanTIMP-1, Rabbit anti-human ICAM-1, Rabbit anti-humanVCAM-1, anti-human p-NF-κB p65/NF-κB p65, and anti-human pIκB-α/IκB-α were purchased from Santa Cruz Biotechnology (Santa Cruz, USA). Matrigel was purchased from Becton Dickinson Company. Anti-CD147 antibody was kindly presented by Cell Engineering Research Center, the Fourth Military Medical University. Anti-β-actin antibody was purchased from Sigma Biotechnology.

### Cell lines

SKOV-3 cell lines, which was originated from ovarian serum cell carcinoma and NIH3T3 fibroblasts, were grown in 1640 (Gibco, USA) supplemented with 100 U/ml penicillin, 100 μg/ml streptomycin, and 10% heat-inactivated FBS (Gibco, USA). Cultures were maintained at 37 °C in a 5% CO_2_/95% air atmosphere.

### Cell Proliferation assay

SKOV-3 cells were seeded in a 96-well plate at 4 × 10^3^cells/well in 200 μL of medium. After 24 h of culture, supernatant was removed and serum–free 1640 containing various concentrations of BDMC was added and incubated for 6 h, 12 h, 24 h. At each time point, 20 μl MTT (5 mg/ml) was added to each well and the cultures were incubated for an additional 4 h at 37 °C. Culture medium was then removed and the formazan crystals dissolved by the addition of DMSO (150 μl/well). Absorbance was measured at 570 nm using an ELISA plate reader. All experiments were performed a minimum of 3 times. Data are presented as the average value ± the standard error of the mean. Cell growth inhibition rate = 1-(experimental group OD value/control group OD value) ×100%.

### Wound healing motility assay

SKOV-3 cells were plated in 12-well plates at 4 × 10^5^ cell/well and cultured in medium containing 10% FBS to near confluence of the cell monolayer. The cells were carefully wounded using a yellow pipette tip, and cellular debris was removed by washing with 1640. The wounded monolayer was incubated with or without BDMC (0 μM, 5 μM, 10 μM and 15 μM) for 24 h in 1640 serum-free medium. Cell migration into the wound area was photographed under phase-contrast microscopy.

### Cell adhesion assay

Briefly, the 96-well plates were coated with 50 μg/ml, 100 μl/well of matrigel, 20 μg/ml, 100 μl/well of Fibronectin, 20 μg/ml, 100 μl/well of BSA and incubated for 24 h at 4 °C. Nonspecific binding sites were blocked with 0.1% bovine serum albumin (BSA) for 1 h at 4 °C, followed by washing three times with phosphate-buffered saline (PBS). The cells were incubated with or without BDMC (5 μM, 10 μM and 15 μM) for 24 h before seeding. The treated cells were trypsinized and resuspensed in1640 serum-free medium; 3 × 10^4^ cells were added to each coated well. The cells were incubated at 37 °C for 1 h and the non-adherent cells were removed by washing with PBS 3 times. MTT dye (15 μl, 5 mg/ml) was added and the plate was incubated for an additional 4 h. Culture medium was then removed and the formazan crystals dissolved by the addition of DMSO (150 μL/well). Absorbance was measured at 570 nm using an ELISA plate reader.

### Cell invasion assay

The invasive behavior of SKOV-3 cells was tested using the modified transwell chamber assay as described previously. Briefly, polyvinylpyrrolidone-free polycarbonate filters (Millipore) (8 μM pore size) were coated with matrigel (15 μg/filter). The medium in the lower chamber contained serum-free culture conditioned medium of NIH3T3 fibroblast cells, which acts as a chemoattractant. SKOV-3 cells (1.5 × 10^5^ cell/chamber) were plated into the upper chamber with or without various concentrations of BDMC and incubated for 24 h at 37 °C, 5% CO_2._ After incubation, the non invading cells were removed from the upper surface of the membrane. The invading cells on the lower surface of the membrane were fixed with methanol for 10 min, stained with toluidine blue for 5 min and washed 3 times with water. The cells that had actively migrated to the under surface of the filter were dissolved with 20% acetic acid and indirectly quantitated by measuring the absorbance at 570 nm. Control experiment was performed in the absence of a chemoattractant.

### Cell cycle analysis

To determine the effect of BDMC on the cell cycle, SKOV-3 cells were treated with BDMC (5 μM, 10 μM, 15 μM) for 6 h, 12 h and 24 h, respectively, then washed, and fixed with 70% ethanol. After incubation overnight at −20 °C, cells were washed with PBS and then suspended in staining buffer (propidium iodide, 10 mg/ml; Tween-20, 0. 5%; RNase, 0. 1% in PBS). The cells were analyzed using a Multicycle for Windows 32-bit (Beckman Coulter, USA). Gating was set to exclude cell debris, cell doublets and cell clumps.

### Apoptotic analysis

Apoptosis was analyzed by using flow cytometry. The cells were incubated with or without BDMC (5 μM, 10 μM and 15 μM) for 24 h. After seeded on a 6-well plate for 24 h, SKOV3 cells were harvested, washed with PBS, and stained with FITC-Annexin V (Sigma) and propidium iodide (PI). Cell apoptosis was analyzed by flow cytometry using CellQuest™ software.

### Western blotting analysis

Cells were washed in PBS and lysed in boiling sodium dodecyl sulfate–polyacrylamide gel electrophoresis (SDS-PAGE) sample buffer [62.5 mM Tris (pH6.8), 1% SDS, 10% glycerol, and 5% β-mercaptoethanol]. The lysates were boiled for 5 min, separated by SDS–PAGE, and transferred to an Immobilon membrane (Millipore). After nonspecific binding sites were blocked for 1 h using 5% skim milk, the membranes were incubated with specific antibodies. Membranes were then washed three times with Tris-Buffered Saline Tween-20 (TBST) and incubated further for 1 h with the corresponding secondary antibodies. Visualization of protein bands was accomplished using ECL (millpore, corporation, USA). The respective protein band intensity was quantified by densitometric analysis using the Gel-pro Analyzer.

### Determination of MMP-2 activity

For quantification of active MMP-2, we introduced the high-sensitivity MMP-2 Activity Biotrak Assay System kit (GE Healthcare), according manufacturer’s instructions. In microtitre wells precoated with anti-MMP-2 antibody that bound active MMP-2, the assay was performed to activate a detection enzyme, which in turn activated a detectable chromogenic substrate. The active MMP-2 levels were expressed as fold increase over the counterpart in control group.

### Measurement of active MMP-9 concentration

The MMP-9 activity was quantified using a human active MMP-9 fluorescent assay kit (F9M00, R&D Systems, Minneapolis, MN, USA). The aminophenylmercuric acetate (APMA, 1 mM), a chemical activator of MMP-9, was added to determine total MMP-9 concentration, including pro-MMP-9. Complementary samples without the addition of APMA measured the amount of endogenously active MMP-9 concentration. The assay sensitivity was 0.005 ng/mL.

### Luciferase reporter gene assay

To assess the promoter activity of NF-κB, cells were transiently cotransfected with p-NF-κB-luciferase vector [containing multiple copies of the NF-κB consensus sequence fused to a TATA-like promoter (P_TAL_)] or with the negative control (p-TAL-luciferase vector), together with the p-CMV-β-galactosidase vector (Promega) using LipofectAMINE 2000 reagent (Invitrogen). By using Luciferase assay system (Promega), the reporter activity was measured and then normalized to β-galactosidase activity.

### Superoxide production quantification

Lucigenin-enhanced luminescence was introduced to measure celluar superoxide content. SKOV-3 cells were lysed and transferred into a polypropylene tube containing 1 mL PBS and lucigenin (Sigma, 0.25 mmol/L). The tube was placed in a FB12-Berthold luminometer (Berthold Technologies, Bad Wildbad, Germany). The RLU emitted was recorded and integrated over 30 sec intervals for 5 min. The activity was normalized with total protein weight.

### Statistical analysis

All statistical analyses were performed using SPSS 16.0 software. Statistical analyses were performed using one-way ANOVA. *P* < 0.05 was considered statistically significant.

## Results

### BDMC inhibited the cell population growth of human ovarian cancer

**Figure 1:**
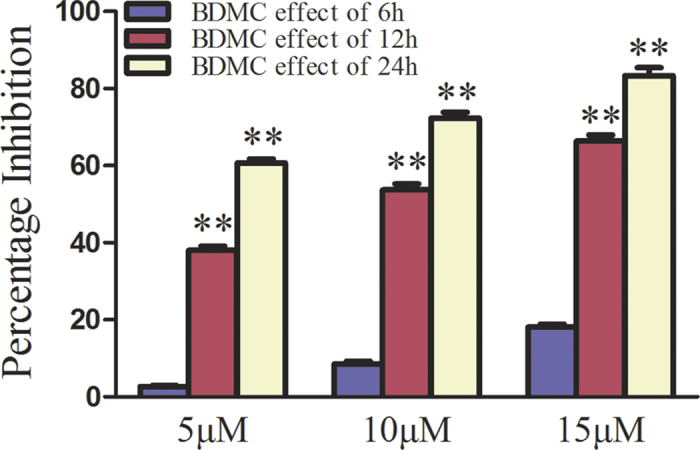
BDMC suppressed the proliferation of SKOV-3 cells in a dose- and time- dependent manner. Cell proliferation was determined by the MTT assay. BDMC, Bisdemethoxycurcumin. The data represent as mean ± SEM. ^**^p < 0.01 *vs*. BDMC effect of 6 h.

Since proliferation effectively reflects the malignancy of tumors, proliferation assessment is an important way to understand the biological behavior of tumors. Using the MTT method, we detected absorbance values (570 nm) of cells at different time points (6 h, 12 h and 24 h) after treatment with various concentrations of BDMC, and their cellular proliferation was monitored. The result demonstrated that BDMC inhibits the cell population growth of human ovarian cancer cell line SKOV-3 (Fig. 1). The anti-proliferative effect of BDMC is in a dose- and time- dependent manner.

### BDMC arrested SKOV-3 cells cycle

As illustrated in Supplemental Table 1, after BDMC treatment, the percentage of cells in the G1 phase significantly increased. BDMC (15 μM, at 24 h) showed a progressive accumulation of cells in G1 phase (79.7%, compared with baseline of 58.1%), accompanied by a decreasing number of cells in S phase (16.4%, compared with baseline of 35.2%) or in G2–M phase (3.89%, compared with a baseline of 6.73%). These results indicate that BDMC can arrest SKOV-3 cells cycle.

### BDMC inhibited the motility of SKOV-3 cells

**Figure 2:**
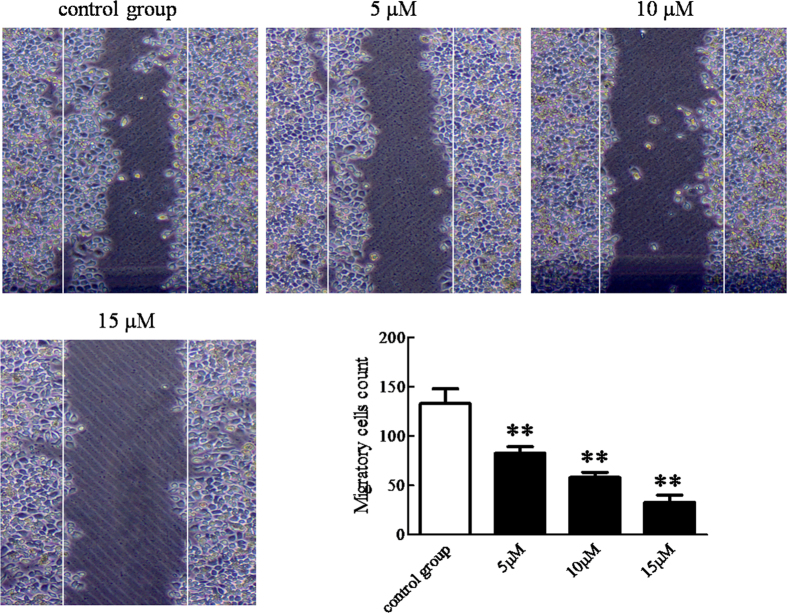
BDMC inhibits the motility of SKOV-3 cells in a dose-dependent manner. For the cell migration assay, cells grew to 60~70% confluence. The cells were carefully wounded using a 20 μl yellow pipette tip in monolayer, resulting in cell wound model. The cellular debris was removed by washing with PBS. The wounded monolayer was incubated with or without BDMC (5 μM, 10 μM and 15 μM) for 24 h in 1640 serum-free medium. Cells were photographed under inverted microscopy. Migratory cells count was analyzed. BDMC, Bisdemethoxycurcumin. The data represent as mean ± SEM. ^**^p < 0.01 *vs*. control group.

Through a visible view in the microscope, thin scratch width of the cell-free zone was observed. After 24 h migration movement, the scratch in control group is basically covered. With the increasing of BDMC concentration, the scratch areas were more evident in those experimental groups (Fig. 2). The results reveal that BDMC significantly inhibits the motility of SKOV-3 cells.

### BDMC inhibited the adhension of SKOV-3 cells

**Figure 3:**
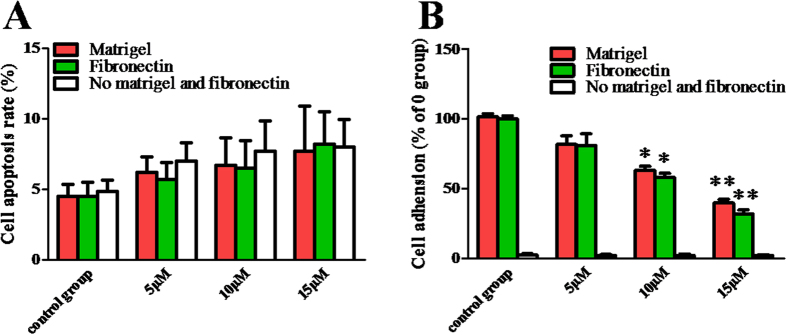
BDMC depressed the adhension of SKOV-3 cells in a dose-dependent manner. (**A**) Cell apoptosis rate was assessed by flow cytometry. (**B**) Matrigel and fibronectin were coated onto the surface of 96-well plates. The coated wells were blocked with BSA for 1 h at 4 °C. SKOV-3 cells treated with the indicated concentration of BDMC for 24 h were collected and added to the ECM-coated plate. After 1 h of incubation, non-adherent cells were removed by washing with PBS for 3 times. MTT dye (15 μl, 5 mg/ml) was added and the plate was incubated for an additional 4 h. Culture medium was then removed and the formazan crystals dissolved by the addition of DMSO (150 μL/well). The absorbance was measured at 570 nm. BDMC, Bisdemethoxycurcumin. The data represent as mean ± SEM. ^*^p < 0.05, ^**^p < 0.01 *vs*. control group.

The adhesion of cancer cells to ECM molecules is the first step of tumor cells invasion. To determine whether BDMC inhibited SKOV-3 cells attachment to matrigel and fibronectin, a cell adhesion assay was performed. First of all, we found that there were not any significant differences in cell viability among all groups (Fig. 3A). And then, we found that BDMC (15 μM, 24 h) treatment significantly reduced cell attachment to matrigel and fibronectin by 58.4% and 50.8%, when compared with control group respectively (Fig. 3B). These results show that BDMC significantly inhibits the adhension of SKOV-3 cells.

### BDMC inhibited the invasion and migration of SKOV-3 cells

**Figure 4:**
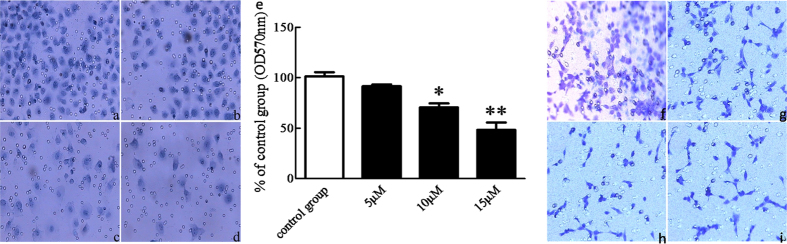
BDMC suppressed the invasion of SKOV-3 cells in a dose-dependent manner. SKOV-3 cells were seeded onto a Matrigel-coated filter containing BDMC with various indicated concentrations (0, 5, 10, 15 μM) for cell invasion assay incubated for 48 h in 37 °C. After 48 h incubation, non-adherent cells were removed, and adherent cells were stained using crystal violet staining. (**A**) control group; (**B–D**) The cell invasion situation with 5 μM, 10 μM, 15 μM of BDMC for 24 h respectively. (**E**) After extensive washing, the stained cells were lysed with 10% acetic acid, and the absorbance was measured at 570 nm. BDMC, Bisdemethoxycurcumin. The data represent as mean ± SEM. ^*^p < 0.05, ^**^p < 0.01 *vs*. control group. BDMC inhibited the migration of SKOV-3 cell. For the cell migration assay, SKOV3 cells were seeded onto a no matrigel-coated filter containing BDMC at the various indicated concentrations (0, 5, 10 and 15 μM) for the cell migration assay, and incubated for 24 h in 37 °C. (**F**) control group; (**G–I**) SKOV3 with BDMC (5, 10, 15 μM). BDMC, Bisdemethoxycurcumin.

During the migration phase of metastasis, cancer cells must pass through the ECM. To evaluate whether BDMC exerts effects on the invasive behavior of cancer cells, we measured the invasive ability of cells by using a matrigel-coated membrane. The cell invasion and migration assay were performed with different BDMC concentrations. As shown in Fig. 4, BDMC significantly decreased the invasion and migration of SKOV-3 cells in a dose-dependent manner.

### BDMC inhibited the extracellular matrix degradation-associated proteins

**Figure 5:**
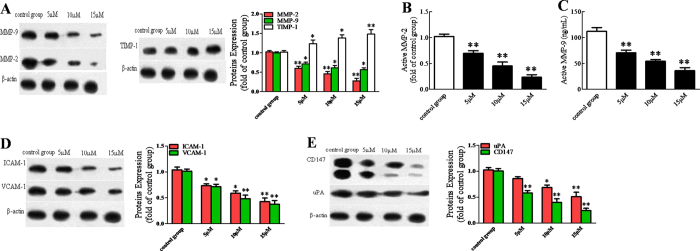
BDMC inhibited the extracellular matrix degradation-associated proteins. (**A,D,E**) The cells were treated with various concentrations of BDMC for 24 h. The cell lysate was prepared and used to analyze expression levels of MMP-2/9, TIMP-1, ICAM-1, VCAM-1, uPA and CD147 using western blot analysis. The intensity of protein bands was quantified by densitomety. (**B,C**) MMP-2/9 activity was determined by Activity Assay Kit, respectively. BDMC, Bisdemethoxycurcumin. The data represent as mean ± SEM. ^*^p < 0.05, ^**^p < 0.01 *vs*. control group.

The tumor metastasis is closely associated with the change of extracellular matrix degradation-associated proteins, thus we evaluated those molecules expressions in our study. As a result, we found that BDMC significantly inhibited expressions of MMP-2/9 and uPA (Fig. 5A,D). With Activity Assay Kits, we found that MMP-2/9 activity also significantly decreased after administration of BDMC in a dose-depend fashion (Fig. 5B,C). It is well known that MMP2/9 can be negatively regulated by TIMP-1 and positively regulated by CD147 respectively, thus we further investigated the expressions of TIMP-1 and CD147. As expected, BDMC significantly increased TIMP-1 content and decreased CD147 expression (Fig. 5A,E). In addition, BDMC obviously decreased expressions of ICAM-1 and VCAM-1 (Fig. 5D). Moreover, all the modulation of BDMC is in a dose-dependent manner. These results show that BDMC exhibits anti-cancer effects through modulating the extracellular matrix degradation-associated proteins.

### BDMC suppressed oxidative stress and subsequently reduced NF-κB transcriptional activity in SKOV-3 cells

**Figure 6:**
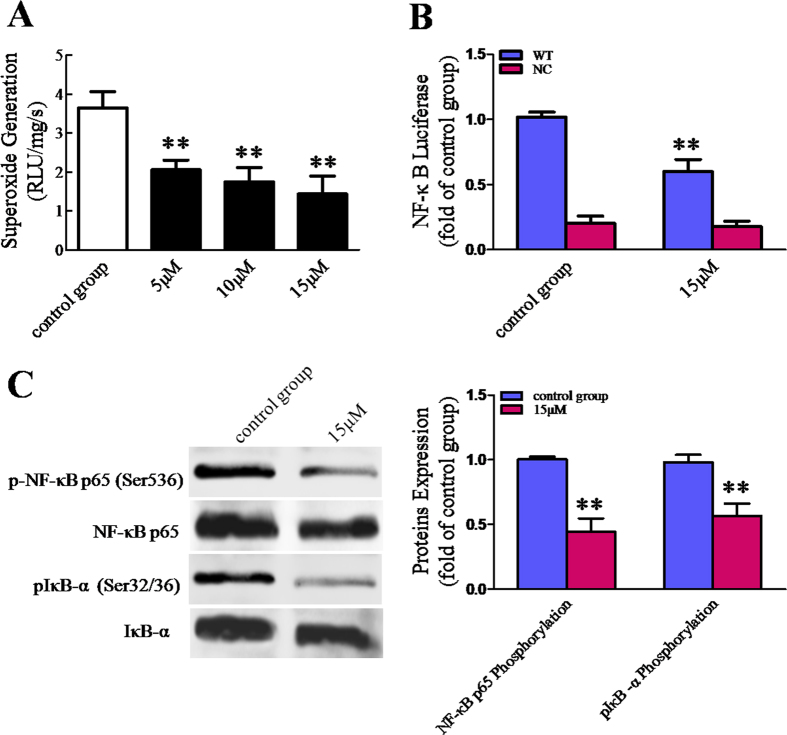
BDMC reduced cellular superoxide generation and NF-κB transcriptional activity. (**A**) Superoxide content was quantified with lucigenin-enhanced luminescence. (**B**) The NF-κB reporter activity was assessed in SKOV-3 cells treated with various concentrations of BDMC, transiently cotransfected with p-NF-κB-luciferase (WT) reporter construct or with negative control (NC) p-TAL-luciferase construct, together with p-CMV-β-galactosidase vector. (**C**) The expression levels of p-NF-κB p65/NF-κB p65 and pIκB-α/IκB-α were evaluated by western blot, in SKOV-3 cells treated with BDMC (0, 15 μM). BDMC, Bisdemethoxycurcumin. The data represent as mean ± SEM. ^**^p < 0.01 *vs*. control group.

To determine the levels of oxidative stress in SKOV-3 cells, we used lucigenin-enhanced luminescence to determine cellular superoxide content. As a result, we found that BDMC obviously reduced superoxide generation in SKOV-3 cells. Moreover, BDMC’s inhibitive effect on oxidative stress was in a dose-depend manner (Fig. 6A). In order to evaluate the ability of BDMC to inhibit NF-κB pathway, we transiently transfected SKOV-3 cells with a luciferase reporter plasmid under the control of a synthetic promoter containing direct repeats of the transcription recognition sequences for NF-κB (p-NF-κB-luciferase) or in parallel to a negative control (p-TAL-luciferase). As suggested in Fig. 6B, BDMC (15 μM) was able to suppress NF-κB-driven luciferase expression. Moreover, we found that BDMC (15 μM) obviously reduced the phosphorylation levels of p65 (Ser536) (Fig. 6C). In NF-κB activation, the phosphorylation of cytoplasmic inhibitor IκB is a key event, leading to p65 release, nuclear translocation and transcriptional activity. Finally, we found that the administration of BDMC (15 μM) markedly reduced IκB-α (Ser32/36) phosphorylation levels (Fig. 6C). These results indicate that BDMC plays anti-cancer effects via inhibiting cellular oxidative stress and subsequently inactivating NF-κB pathway.

## Discussion

Accumulative evidences reveal that there are 78% Curcumin (Cur), 16% Demethoxycurcumin (DMC) and 5% BDMC in curcuminoids^10^, among which DMC or BDMC show higher potency than Cur^11^. Consistent with previous study^12^,^13^, our work also revealed that BDMC is the most active form for modulation of MDR-1 gene. There are several complex processes in tumor invasion and metastasis, including cell adhesion, proteolytic ECM degradation, cell migration to basement membranes, and remigration and growth of tumor at metastatic sites, etc.^14^, suggesting the potential therapeutic approaches targeting at ECM degradation. Although, the anti-invasive property of BDMC has been reported, there is no direct evidence to show that BDMC exerts its anti-invasion and -metastasis effects in ovarian cancer through modulation of ECM.

In the present study, we found that BDMC at a low-and non-cytotoxicity dose markedly inhibited the invasion and metastasis of SKOV-3 cells by influencing the degradation of extracellular matrix and basement membrane ingredients, which is in line with previous reports^15^,^16^. There are generally three aspects underlying the inhibition of BDMC on tumor cell invasion and metastasis: 1) At cellular level, BDMC suppressed cell cycle. As illustrated in Supplemental Table 1, different concentrations of BDMC (0, 5 μM, 10 μM, 15 μM) gradually increased G1 phase proportion of SKOV-3 cells (6 h, 12 h, 24 h), indicating the uncoordinated growth. This study demonstrated that BDMC can arrest SKOV-3 cells in G1 phase. 2) At molecular level, BDMC inhibited expressions of MMPs and urokinase, revealing the inhibition of ECM degradation and consequently tumor invasion. As proteolytic enzymes, MMPs secretion can be enhanced by vascular endothelial growth factor (VEGF) and other angiogenic factors. TIMPs are able to inhibit the activity of MMPs to prevent tumor cell invasion and metastasis. Under normal conditions, MMPs/TIMPs system remains balanced to maintain matrix synthesis and catabolism. Once there are certain factors breaking MMPs/TIMPs ratio, matrix degradation and tumor cell invasion will happen^10^. In addition, CD147 is also known to induce MMPs expression^17^. Several studies claim that high expression levels of uPA^18^ and MMP are closely correlated with the invasive capacity of breast cancer cells^19^. Consistently, our data supported the inhibitory effect of BDMC on cancer invasion by reducing expressions of uPA and MMPs to restrict the degradation of ECM. 3) In addition, elevated expressions of ICAM-1 and VCAM-1 have been reported in lung^20^,^21^, gastric and breast cancer tissues^22^, with highest levels in samples from patients with metastasis. In our study, BDMC inhibited the expressions of ICAM-1 and VCAM-1 in a dose-dependent manner. Moreover, in the present study, we found that BDMC also reduced oxidative stress in a dose-dependent manner, revealing that oxidative stress may mediate BDMC’s suppressive effects on the degradation of ECM. To evaluate the influence of BDMC on NF-κB pathway, we transiently transfected SKOV-3 cells with a luciferase reporter plasmid under the control of a synthetic promoter containing direct repeats of the transcription recognition sequences for NF-κB or a negative control. We found that BDMC was able to suppress NF-κB-driven luciferase expression, revealing the inactivation of NF-κB pathway. As a complex of proteins, NF-κB includes subunits of p50 and p65. The phosphorylation of p65 is required for NF-κB transcriptional activity. Moreover, in NF-κB activation, the phosphorylation of cytoplasmic inhibitor IκB is a key event, leading to p50/p65 heterodimer release, nuclear translocation and transcriptional activity. In the end, we found that the administration of BDMC markedly reduced the phosphorylation levels of p65 (Ser536) and IκB-α (Ser32/36). These results indicate that BDMC is able to prevent the activation of NF-κB pathway in SKOV-3 cells. Consistent with our findings, many studies confirm that MMPs secretion can be influenced by oxidative stress^3^,^4^, and NF-κB signaling can be altered by ROS generation^5^.

In conclusion, our data indicate that BDMC is able to inhibit adhesion, invasion and migration of human ovarian cancer cells. Further studies indicate that BDMC reduces expressions of invasion-associated proteins via suppressing oxidative stress and subsequently inactivating NF-κB pathway in SKOV-3 cells.

## Additional Information

**How to cite this article**: Pei, H. *et al*. Bisdemethoxycurcumin inhibits ovarian cancer via reducing oxidative stress mediated MMPs expressions. *Sci. Rep.* **6**, 28773; doi: 10.1038/srep28773 (2016).

## Supplementary Material

Supplementary Information
