# Supplementary material for: Bisdemethoxycurcumin inhibits ovarian cancer via reducing oxidative stress mediated MMPs expressions
Source: Sci Rep. 2016 Jun 28;6:28773. doi: 10.1038/srep28773 (PMC4923879; doi:10.1038/srep28773)
Supplement: Supplementary Information [file srep28773-s1.pdf]

Bisdemethoxycurcumin inhibits ovarian cancer via reducing oxidative stress mediated MMPs expression

Haifeng Pei[1], Yi Yang[1], Lin Cui[2], Jiong Yang[1], Xiuchuan Li[1], Yongjian Yang[1,*], Haixia Duan[3,*]

[1] Department of Cardiology, Chengdu Military General Hospital, Chengdu 610083, China;

[2] Department of Orthopedics, Chengdu Military General Hospital, Chengdu 610083, China;

[3] Department of Obstetrics and Gynecology, Affiliated Guangren Hospital, College of Medicine, Xi’an Jiaotong University, Xi’an 710004, China.

Running title: BDMC and Ovarian Cancer

[*]**Address correspondence to:**

Haixia Duan, MD, PhD
Affiliated Guangren Hospital,
College of Medicine, Xi’an Jiaotong University
21 Jiefang Road
Xi’an 710004, China
E-mail: dhx20167@126.com
Tel: +86-29-8742-0006
Fax: +86-29-8742-0008
Or
Yongjian Yang, MD, PhD
Chengdu Military General Hospital
270 Tianhui Road
Chengdu 610083, China
E-mail: yyj10001@126.com
Tel: +86-28-8657-0211
Fax: +86-28-8357-2211

Supplemental Table 1. The influence of BDMC on the cycle of SKOV-3 cell. BDMC arrests the cells at G1 phase of the cell cycle. Serum-1640 SKOV-3 (1×10^6cells/ml) was incubated in the absence or presence of different doses of BDMC for 6, 12 and 24h, respectively. Thereafter, the cells were washed, fixed, stained with propidium iodide, and analyzed for DNA content by flow cytometry. BDMC, Bisdemethoxycurcumin. $^{**}p<0.01$ *vs*. 0 μM BDMC.

Table 1. The influence of BDMC on the cycle of SKOV-3 cell.

BDMC (concentration) (μM)	Action time (h)	Proportion of cell cycle (%)		
		G1	S	G2
—	0	58.1	35.2	6.73
5	6	60.5**	32.2	7.29
10	6	65.6**	22	7.94
15	6	66.5**	26.7	6.72
5	12	70.1**	21.6	8.27
10	12	71.7**	20.1	8.16
15	12	75.4**	19.7	4.86
5	24	76.7**	17.3	6.03
10	24	78.5**	15.6	5.85
15	24	79.7**	16.4	3.89
